# Altered White-Matter Microstructure in Conduct Disorder Is Specifically Associated with Elevated Callous-Unemotional Traits

**DOI:** 10.1007/s10802-017-0375-5

**Published:** 2017-12-22

**Authors:** Ignazio Puzzo, Kiran Seunarine, Kate Sully, Angela Darekar, Chris Clark, Edmund J. S. Sonuga-Barke, Graeme Fairchild

**Affiliations:** 1 Forensic Research & Development Domain, Broadmoor High Secure Hospital, West London Mental Health Trust, Southall, UK; 2 Developmental Imaging and Biophysics Section, UCL Institute of Child Health, London, UK; 3 Manchester, UK; 4 Department of Medical Physics, University Hospital Southampton NHS Foundation Trust, Southampton, UK; 5 Institute of Psychology, Psychiatry and Neuroscience, Kings College London, London, UK

**Keywords:** Conduct disorder, Callous-unemotional traits, Brain structure, Diffusion tensor imaging, Structural connectivity

## Abstract

Adolescents with conduct disorder (CD) and elevated callous-unemotional (CU) traits have been reported to present with a more severe and persistent pattern of antisocial behaviour than those with low levels of CU traits. However, relatively few studies have investigated whether there are differences in brain structure between these subgroups.We acquired diffusion tensor imaging data and used tract-based spatial statistics (TBSS) to compare adolescents with CD and high levels of CU traits (CD/CU+; *n* = 18, CD and low levels of CU traits (CD/CU-; *n* = 17) and healthy controls (HC; *n* = 32) on measures of fractional anisotropy (FA), axial (AD), radial (RD) and mean (MD) diffusivity. Compared to CD/CU- adolescents, those with CD/CU+ presented increased FA and reduced RD and MD (lower diffusivity) in several tracts including: body and splenium of the corpus callosum, right inferior longitudinal fasciculus, ILF; right inferior fronto-occipital fasciculus, IFOF; left superior longitudinal fasciculus, SLF; left cerebral peduncle, bilateral internal capsule, left superior and posterior corona radiata, bilateral thalamic radiation and left external capsule. In addition, relative to CD/CU- individuals, adolescents with CD/CU+ showed lower diffusivity (indexed by reduced RD and MD) in left uncinate fasciculus and bilateral fornix. Finally, relative to healthy controls, CD/CU+ individuals showed lower diffusivity (reduced RD) in the genu and body of the corpus callosum and left anterior corona radiata. These results suggest that CD/CU+ individuals present with white-matter microstructural abnormalities compared to both CD/CU- individuals and age-matched healthy controls. This finding is consistent with emerging evidence suggesting that CD/CU+ represents a distinct subtype of CD, and illustrates the importance of accounting for heterogeneity within CD populations.

## Introduction

Conduct Disorder (CD) is characterized by pervasive and persistent antisocial behavior that violates other people’s rights or age-appropriate societal norms (APA 2013). It affects around 5% of the adolescent population (NICE 2006), and is associated with a range of negative educational, social and mental health outcomes. Most importantly, it is a precursor of adult antisocial personality disorder (Teplin et al. 2005; Fazel et al. 2008). The fifteen symptoms that make up the CD diagnostic criteria fall into four distinct categories: ‘aggression to people and animals’, ‘destruction of property’, ‘deceitfulness or theft’, and ‘serious violations of rules’ (APA 2013). Given that over 32,000 different symptom profiles could lead to a CD diagnosis (Nock et al. 2006), the CD population is highly heterogeneous. It has been argued that one important differentiating characteristic relates to the degree to which the individual displays guilt, empathy and other prosocial emotions. These characteristics, often considered under the umbrella term of callous-unemotional (CU) traits (Kimonis et al. 2006) have been shown to distinguish between subgroups of CD individuals that differ in terms of etiology and neurocognitive processing (Frick and Viding 2009).

Functional magnetic resonance imaging (fMRI) studies have provided evidence that individuals with conduct problems and high levels of CU traits (CD/CU+) process emotional stimuli differently from those with conduct problems and low levels of CU traits (CD/CU-). Reduced amygdala responses to fearful facial expressions have been reported in adolescents with CD or conduct problems, and elevated CU traits compared to typically-developing controls (Jones et al. 2009; Marsh et al. 2008), whereas increased amygdala responses to fearful faces have been demonstrated in children with conduct problems and low levels of CU traits compared with their high CU traits counterparts (Viding et al. 2012). Recent structural MRI studies have also provided evidence that alterations in grey matter volume may be more pronounced in CD/CU+ individuals relative to their CD/CU- counterparts (De Brito et al. 2009; Fairchild et al. 2013; Sebastian et al. 2016). These findings suggest that these subgroups may differ in brain function and structure, but an open question is whether they also differ in terms of structural connectivity.

Diffusion Tensor Imaging (DTI) is a non-invasive magnetic resonance imaging (MRI) technique that enables the measurement of diffusion of water molecules along anisotropic fiber bundles. Generally, DTI studies have used fractional anisotropy (FA) as a general index of white-matter microstructural “integrity”. FA represents the proportion of diffusion in the direction parallel to the axon bundle (axial diffusivity, AD) relative to the direction perpendicular to the axon bundle (radial diffusivity, RD). FA is known to be influenced by both the density and dispersion of axonal fibres. Alongside FA are measures of axial and radial diffusivity (AD and RD), which reflect the magnitude of diffusion along the dominant orientation of the fibers and perpendicular to the dominant orientation of the fibers, respectively. There is evidence that CD is associated with abnormalities in white-matter microstructure, although the literature is somewhat inconsistent in terms of the location and direction of findings (Waller et al. 2017).

A number of studies have reported increased FA in male adolescents with CD (Passamonti et al. 2012; Sarkar et al. 2013; Zhang et al. 2014a), suggesting general changes in microstructural properties. Using tractography, Sarkar et al. (2013) found that individuals with CD had significantly increased FA and reduced RD in the left UF compared with healthy controls (HCs). The UF is a key white-matter tract that connects the anterior temporal lobe (including the amygdala) with the orbitofrontal cortex (Catani et al. 2013). Passamonti et al. (2012) used Voxel-based Diffusion Tensor Imaging (VB-DTI) and showed that adolescents with CD displayed higher FA within the right external capsule. The authors followed up this finding using tractography, and demonstrated that this effect was primarily driven by increased FA within the UF. Zhang et al. (2014a) found significantly higher FA and lower RD in the bilateral UF in males with CD compared to controls. The same group also reported increased FA in males with CD predominantly in the corpus callosum, bilaterally, and in the left anterior corona radiata (ACR) and right superior corona radiata (SCR) tracts (Zhang et al. 2014a). In addition, individuals with CD showed lower RD in bilateral corpus callosum. A recent study showed that callous-unemotional traits in adolescent arrestees were positively associated with FA and AD in specific white matter tracts, including the corpus callosum and corticospinal tract (Pape et al. 2015). Along the same lines Sarkar et al. (2016) reported a positive correlation between FA and callous unemotional traits in right posterior limb of the internal capsule, superior longitudinal fasciculus, corticopontocerebellar tract; and left cerebellar white matter.

In contrast to the above reports, some studies have reported reduced FA in CD samples. Using tract-based spatial statistics (TBSS), a method of assessing changes in white-matter microstructure on a voxel-wise level across the whole brain, Wang et al. (2012) found significantly lower FA and higher mean diffusivity (MD), RD and AD in many white-matter tracts in adolescents with disruptive behavior disorders (DBDs) and comorbid ADHD compared to HCs (Wang et al. 2012). Haney-Caron et al. (2014) reported lower FA and AD in the anterior/superior corona radiata and the inferior longitudinal and fronto-occipital fasciculi in a mixed-sex group of adolescents with non-comorbid CD (Haney-Caron et al. 2014). Recently, a DTI study by Breeden et al. (2015) found that CU traits were negatively correlated with FA values in the bilateral uncinate fasciculus (UF) and stria terminalis/fornix.

We propose that one important factor responsible for the inconsistent findings described above is the varying levels of CU traits in CD samples. To date only a few studies of CD youth included measures of CU or psychopathic traits, which limits the extent to which they were able to evaluate whether these traits accounted for white-matter tract microstructural differences. Of these studies, one study that examined a community sample of youth found that CU traits were specifically related to lower FA in the UF and fornix (Breeden et al. 2015). However, three studies that investigated CU/psychopathic traits found that they were related to higher FA, higher AD and lower RD in various commissural, projection, and association tracts (Pape et al. 2015; Sarkar et al. 2013; Sarkar et al. 2016). CU traits were also found to be specifically related to lower RD and MD of the right UF among males with CD (Zhang et al. 2014a). Alongside CU traits (which are suggested to distinguish between subtypes of CD individuals) other factors such as the presence of co-morbid ADHD (Konrad and Eickhoff 2010; Wang et al. 2012), IQ (Moffitt 1993), age/pubertal stage (Asato et al. 2010), and substance use (Bava et al. 2013) could have contributed to the divergent findings.

ADHD is also associated with abnormalities in structural connectivity (Konrad and Eickhoff 2010). Wang et al. (2012) found that adolescents with DBDs and comorbid ADHD had significantly lower FA, and higher MD, RD and AD in many white-matter tracts compared to controls or those with DBDs alone. Similarly, IQ may be a confounding factor, as CD is robustly associated with lower-than-average IQ (Moffitt 1993). It is therefore probable that the CD and control groups will differ in IQ. Other potential confounding factors include: substance use and age /pubertal stage of the sample. Cross-sectional and longitudinal studies have reported that substance use is associated with alterations in white matter anisotropy and diffusivity (Bava et al. 2013). Although white-matter tracts show increased development with increasing chronological age, most white-matter tracts only reach mature levels of integrity after puberty (Asato et al. 2010). To address these issues in the present study, ADHD symptoms, IQ, age, pubertal stage and substance use will be included as covariates in order to control for their effects.

The present study investigated the impact of CU traits on white-matter microstructural properties in CD adolescents, by explicitly comparing individuals with CD and elevated callous-unemotional traits (CD/CU+), individuals with CD and lower levels of callous-unemotional traits (CD/CU-) and healthy control groups using a similar design to that adopted in earlier fMRI and sMRI studies (e.g., Viding et al. 2012; Sebastian et al. 2016). Furthermore, a correlational design approach investigating relations between CU traits in the CD sample and FA, AD, RD and MD was also performed in order to confirm the sensitivity of the results given possible limitations of group-based approaches.

Based on previous findings, we hypothesized that we would find white-matter microstructural abnormalities in several tracts including, but not limited to, the UF in CD compared to HC participants. In addition to testing for alterations in FA, we used TBSS to investigate the white-matter microstructural variables that give rise to FA – i.e., RD, MD and AD – to understand what might be driving any observed group differences in FA. Moreover, given evidence that CD/CU+ is more severe and persistent than CD/CU- (Rowe et al. 2010; Frick et al. 2014), it was predicted that the former group would display more pronounced white-matter microstructural abnormalities than the latter.

## Materials and Methods

### Participants

Thirty-three male adolescents with CD and 29 male age-matched healthy controls participated in the study. All participants were aged between 13 and 18 years. Healthy control participants were recruited from mainstream schools and colleges. Participants with CD were recruited from Pupil Referral Units and Youth Offending Teams in Southampton and Hampshire. Participants at schools and colleges were contacted through the school/college by sending an information pack about the study to their homes. A member of staff at pupil referral units and youth offending teams described the study to potential volunteers and asked them whether they were interested in taking part. If they were interested in participating, they either sent back reply slips or gave permission for the staff member to pass on their contact details to the research team. Once reply slips or contact details were received, a visit to the participants’ homes to carry out separate semi-structured diagnostic interviews with them and their primary caregiver was arranged. The study was approved by the University of Southampton’s Ethics Committee and Research Governance Office. Written informed consent was obtained from all participants and their parents.

The presence of CD, as well as other common mental health disorders, was assessed using separate interviews with all participants and their parent/carer using the Schedule for Affective Disorders and Schizophrenia for School-Age Children-Present and Lifetime version (K-SADS-PL) (Kaufman et al. 1997). Participants were also assessed for IQ using the two-subtest version of the Wechsler Abbreviated Scale of Intelligence (WASI; Wechsler 1999). The self-report version of the Inventory of Callous-Unemotional traits (ICU) questionnaire was used to assess CU traits in our participants (Essau et al. 2006). The Pubertal Development Scale (PDS) (Petersen et al. 1988) was used to assess participants’ pubertal stage and the Personal Experience Screening Questionnaire (PESQ) (Winters, 1992) was administered to assess substance use.

### Magnetic Resonance Imaging

MR imaging was performed on a Siemens Avanto 1.5 T MRI scanner, using a 12 channel phased array head coil. The MR imaging protocol consisted of a whole brain T1-weighted structural MRI scan and a DTI acquisition. The T1-weighted volume images were acquired using a magnetisation-prepared rapid gradient echo (MPRAGE) sequence with the following parameters: TR/TE/TI = 2400/3.42/1000 ms, flip angle = 80, voxel size = 1.2 mm × 1.2 mm × 1.2 mm, 160 slices (acquired in a sagittal plane), acquisition time = 7 min 42 s.

The DTI (single shot echo planar imaging) sequence parameters were as follows: TR/TE = 8900/109 ms, 63 non-collinear diffusion directions (including 3 interleaved b = 0 acquisitions), voxel size = 2.3 mm × 2.3 mm × 2.3 mm, b-value = 1000 s/mm^2^, parallel imaging factor = 2, 60 slices (acquired in an oblique axial plane avoiding the orbits where possible), acquisition time = 10 min 7 s. The T1-weighted volume images were clinically reported for any structural abnormalities by an experienced pediatric neuroradiologist.

### Data Pre-Processing and Analysis

Raw diffusion weighted imaging data (DICOM images from the scanner) were converted to 4D NIfTI format for each participant*.* The resulting 4D images were visually inspected for artefacts by an experienced rater who was blind to group status. Five datasets were excluded from the analysis due to excessive head motion or signal loss. This left 33 CD participants and 29 healthy controls with usable data. Datasets were then corrected for motion and eddy current distortion using the “eddy” tool, available in FSL (Smith et al. 2004; Smith et al. 2007). Dropout caused by subject motion was corrected by replacing outliers with predictions made by the Gaussian Process used by the eddy tool (Andersson et al. 2016). FA, MD, AD, and RD images were produced by fitting a tensor model at each voxel to the corrected diffusion data using dwi2tensor, part of MRTrix. Tract-based spatial statistics (TBSS) was used to perform voxel-wise statistical analysis of FA, MD, AD, and RD (Smith et al. 2007).

All participants’ FA maps were aligned to the FMRIB58_FA template using a nonlinear registration and then merged into a single 4D image. Next, the mean FA image was created and the tracts were narrowed to generate a mean FA skeleton which represents the centres of all tracts common to all subjects. An FA threshold of 0.2 was chosen to discard non-white matter voxels. The area surrounding the skeleton in each subject’s aligned FA map was searched perpendicular to the skeleton voxel and the locally highest FA values were projected onto the skeleton. This ensures that each subject’s skeleton remains in the group space while representing the centers of each individual’s own unique fiber tracts. We used the projection vectors that were estimated for each subject FA skeleton onto the mean FA skeleton to create MD, AD, RD. The resulting individual skeletonised images were fed into voxel-wise statistical tests (Smith et al. 2007).

In order to assess the impact of CU traits on these parameters, we split the CD sample into higher (CD/CD+) and lower levels of CU traits (CD/CU-) using the group’s median score on the ICU (the total score was used, with scores ranging from 13 to 52; median score was 32). This approach has been used in several previous studies (Sebastian et al. 2012; Viding et al. 2012; Hodsoll et al. 2014). This meant that there were 3 groups: CD/CU+ participants (*n* = 17; mean age 16.1, SD 1.2 yrs), CD/CU- participants (*n* = 16; mean age 16.3, SD 1.5 yrs) and HC participants (*n* = 29; mean age 16.1, SD 1.02; see Table 1 for a summary). Internal validation of the ICU data was performed by examining the relation between total ICU scores and CD symptoms in our CD sample. Results showed a significant and strong positive association between total ICU scores and CD symptoms (r (33) = .602, *p* < 0.05).

The general linear model tool in FSL was used to compare the three groups (CD/CU+, CD/CU- and HC). Age, IQ, ADHD symptoms, and PDS and PESQ scores were entered into the model in order to control for their effects on white-matter microstructural variables.

Permutation-based statistical analysis (5000 permutations) was achieved with *Randomise* implemented in FSL, utilizing threshold-free cluster enhancement method (TFCE) (Smith and Nichols 2009). Statistical maps were then thresholded at *p* < 0.05, whole-brain correction for multiple comparisons (family-wise error [FWE]-corrected). The relevant t contrasts in the Randomise analysis were: CD > HC, HC > CD; these contrasts compared the CD group as a whole (collapsing across CD/CU+ and CD/CU- subjects) versus HCs on FA, MD, AD and RD measures. The additional contrasts tested for differences between the CD/CU+, CD/CU- and HC groups in FA, MD, AD and RD values. In addition, we examined for correlations between a continuous measure of CU traits and FA, AD, RD and MD in order to test the reliability and validity of the group-based analysis described above. Simple effects of callous-unemotional traits were tested in a general linear model (GLM) with age, IQ, ADHD symptoms, and PDS and PESQ scores added as covariates of no interest. We tested for local associations (both positive and negative) between callous-unemotional traits and FA utilizing threshold-free cluster enhancement (TFCE) (Smith and Nichols 2009). Statistical *p*-value maps for each contrast were calculated over 5000 random permutations with a significance threshold of *p* < 0.05. The same procedure was followed for the RD, AD and MD maps.

The most likely anatomical localizations for each cluster showing significant between-group differences in FA, MD, AD, and RD were determined using the JHU_ICBM-DTI-81 White Matter Labels available within the FSL atlas tool (Mori et al. 2006; Wakana et al. 2007; Hua et al. 2008). All statistical analyses pertaining to demographic and clinical characteristics were carried out in SPSS v.21 (IBM Corp., 2012, Armonk, NY, USA).

## Results

A one-way ANOVA revealed group differences in CU traits [F (2, 59) = 28; *p* < 0.0001], IQ [F (2, 59) = 5.8; *p* < 0.005], ADHD symptoms [F (2, 59) = 36.9; *p* < 0.0001 and substance abuse [F (2, 59) = 6.50; *p* < 0.001], but not age [F (2, 59) = 0.19; *p* = 0.82] or pubertal development (PDS) [F (2, 59) = 1.66; *p* = 0.19] (see Table 1). Bonferroni corrected post-hoc comparisons revealed that the CD/CU+ group had significantly higher levels of CU traits than the HC (*p* < 0.0001) and CD/CU- groups (p < 0.0001). Critically, the CD/CU- group did not differ from the HC group in CU traits (*p* = 0.76). The CD/CU+ and CD/CU- groups both had lower IQs than healthy controls (*p* < 0.01 and *p* < 0.05 respectively). However, the CD/CU+ and CD/CU- groups did not differ in IQ (*p* = 1.00) or the number of ADHD symptoms endorsed (*p* = 0.56). However, both the CD/CU+ and CD/CU- groups had significantly more ADHD symptoms than the HC group (*p* < 0.001 in both cases). Similarly, the CD/CU+ and CD/CU- groups did not differ in terms of substance use (p = 0.56), but both groups were significantly higher in terms of substance use than the HC group (*p* < 0.005 in both cases). A Chi-square test showed no significant group differences in handedness (*X* ^2^ (2) = 0.04, *p* = 0.97; see Table 1).

**Table 1:** Sample descriptive and clinical characteristics

Measure	CD/CU + (*n* = 17)	CD/CU - (*n* = 16)	HC (*n* = 29)	*p*-value	Post-hoc tests
Age (years)	*16.2 (1.2)*	16.3 (1.5)	16.2 (1.1)	0.93	–
Estimated IQ	93.7 (10.9)	93.6 (12.5)	102.7 (10.1)	0.005	CD/CU+ = CD/CU-; HC > CD/CU+; HC > CD/CU-
CD symptoms	9.2 (1.6)	7.7 (2.3)	0.2 (0.5)	<0.0001	CD/CU+ > CD/CU- > HC
CU traits (ICU)	38.6 (5.3)	26 (5)	23.4 (8.2)	<0.0001	CD/CU+ > CD/CU-; CD/CU+ > HC; CD/CU- = HC
ADHD symptoms	8.4 (4.5)	7.2 (4.5)	0.5 (1.1)	<0.0001	CD/CU+ = CD/CU-; CD/CU+ > HC; CD/CU- > HC
PESQ	36.9 (16.8)	36.3 (11.7)	25.2 (9.4)	0.003	CD/CU+ = CD/CU-; CD/CU+ > HC; CD/CU- > HC
PDS	5 (4.7)	5.4 (4.6)	7.8 (6.4)	0.19	–
Handedness	15 (right); 2 (left)	14 (right); 2 (left)	25 (right); 4 (left)	Chi-square	*X* ^2^ (2, 62) = 0.04, p = 0.97

Means are presented with Standard Deviations in parentheses. ADHD = Attention-Deficit/Hyperactivity Disorder; CD = Conduct Disorder; CD/CU+ = Conduct Disorder with high levels of callous unemotional traits; CD/CU- = Conduct Disorder with low levels of callous unemotional traits; CU = callous-unemotional; HC = Healthy Controls; IQ = Intelligence Quotient; ICU = Inventory of Callous-Unemotional traits, PESQ = Personal Experience Screening Questionnaire; PDS = Pubertal Developmental Scale

## Group-Based Analysis

When treating the CD group as a whole (collapsing across CD/CU+ and CD/CU- participants), there were no significant differences between the CD and the control groups in any of the white-matter microstructural parameters. There were also no significant differences in these microstructural parameters between the CD/CU- and control groups. However, white-matter microstructural parameters in the CD/CU+ group differed significantly from both the CD/CU- and the healthy control groups in several major tracts.

**Fig. 1:**
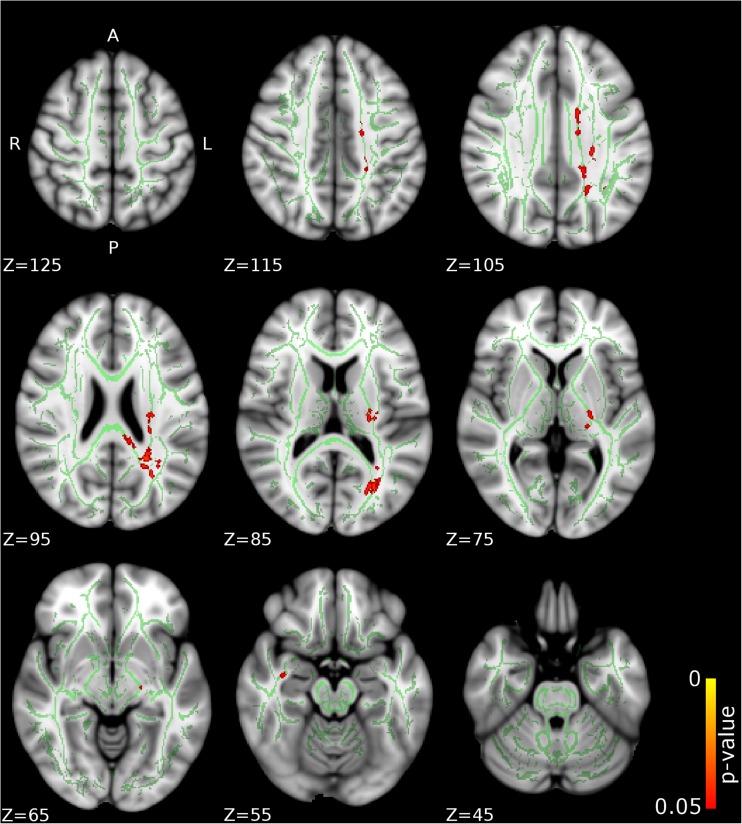
**Differences in fractional anisotropy (FA) between the CD/CU+ and CD/CU- groups.** The figure depicts increased fractional anisotropy in adolescents with Conduct Disorder with elevated callous-unemotional traits (CD/CU+) relative to those with Conduct Disorder with lower levels of callous unemotional traits (CD/CU-). The axial images are thresholded at *p* < 0.05, threshold-free cluster enhancement (TFCE) correction, i.e., correction for all voxels in the white-matter skeleton. Significance level of the findings is indicated by the red-yellow color scheme (with the most significant findings shown in yellow). All findings are superimposed on the mean FA background image and the outline of the TBSS-generated white-matter tract skeleton, shown in green

**Fig. 2:**
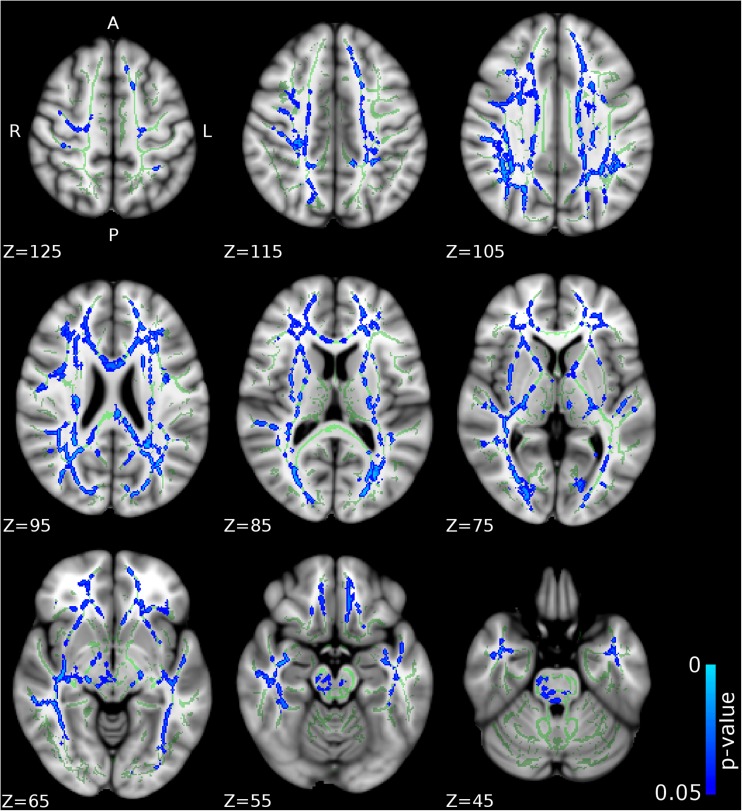
**Differences in radial diffusivity (RD) between the CD/CU+ and CD/CU- groups.** The figure displays regions that showed reduced radial diffusivity in adolescents with Conduct Disorder with elevated callous-unemotional traits (CD/CU+) relative to those with Conduct Disorder with lower levels of callous unemotional traits (CD/CU-). The axial images are thresholded at p < 0.05, threshold-free cluster enhancement (TFCE) correction, i.e., correction for all voxels in the white-matter skeleton. Significance level of the findings is indicated by the blue color scheme (with the most significant findings shown in light blue). All findings are superimposed on the mean FA background image and the outline of the TBSS-generated white-matter tract skeleton, shown in green

**Fig. 3:**
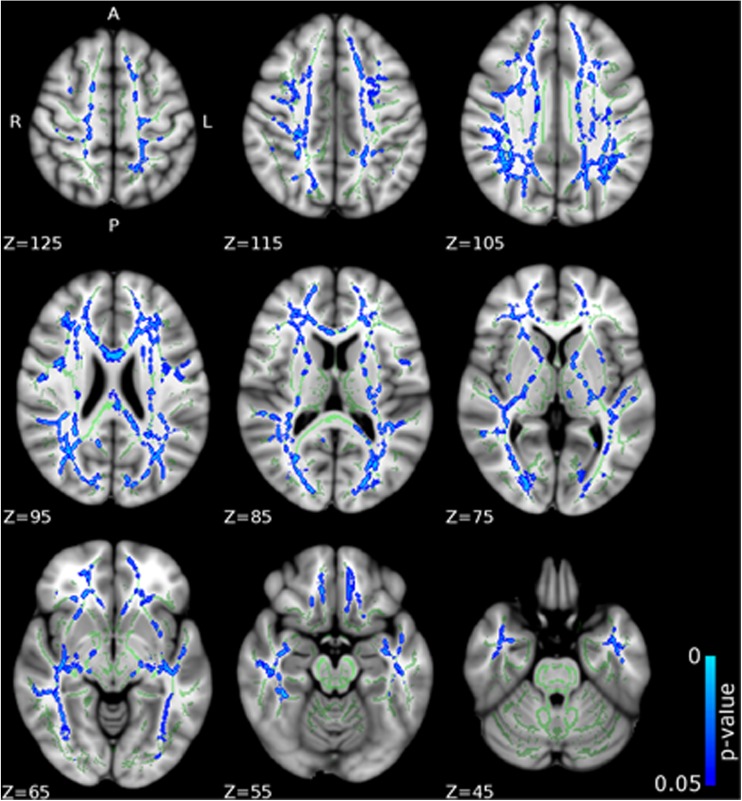
**Differences in mean diffusivity (MD) between the CD/CU+ and CD/CU- groups.** The figure displays regions that showed reduced mean diffusivity in adolescents with Conduct Disorder with elevated callous-unemotional traits (CD/CU+) relative to those with Conduct Disorder with lower levels of callous unemotional traits (CD/CU-). The axial images are thresholded at p < 0.05, threshold-free cluster enhancement (TFCE) correction, i.e., correction for all voxels in the white-matter skeleton. Significance level of the findings is indicated by the blue color scheme (with the most significant findings shown in light blue). All findings are superimposed on the mean FA background image and the outline of the TBSS-generated white-matter tract skeleton, shown in green

Compared to CD/CU- individuals, adolescents with CD/CU+ showed higher FA and lower RD and MD (indexing lower diffusivity) in commissural tracts (body and splenium of the corpus callosum), association tracts (right inferior longitudinal fasciculus, ILF; right inferior fronto-occipital fasciculus, IFOF; left superior longitudinal fasciculus, SLF) and projection/thalamic pathways (left cerebral peduncle, bilateral internal capsule, left superior and posterior corona radiata, bilateral thalamic radiation and left external capsule). Relative to the CD/CU- group, the CD/CU+ group also had lower RD and MD in most of the tracts mentioned above (see Figs. 1, 2, 3 and Table 2). Compared to the CD/CU- group, the CD/CU+ group also presented with lower RD and MD in left uncinate fasciculus and bilateral fornix (see Figs. 2 and 3 and Table 2).

**Table 2:** Tracts showing significant group differences in FA, AD, RD and MD

**Contrast**	White matter label	Hemisphere	Cluster size	Peak x,y,z			p-value
CD/CU + > CD/CU -	**FA**						
	BCC	–	94	104	128	104	0.043
	SCC	–	196	99	88	89	0.05
	PIC	L	126	113	107	76	0.046
	RIC	L	10	114	106	76	0.047
	SCR	L	212	118	107	91	0.043
	PCR	L	257	115	102	100	0.043
	PTR	R	13	51	80	71	0.05
		L	98	118	58	85	0.042
	SS	R	12	50	89	68	0.047
	EC	L	36	118	107	86	0.043
CD/CU + < CD/CU –	**MD**						
	GCC	–	636	96	146	90	0.014
	BCC	–	1254	95	145	91	0.014
	SCC	–	422	105	84	95	0.013
	CP	R	71	76	117	64	0.035
		L	60	104	102	64	0.016
	AIC	R	241	68	133	87	0.025
		L	244	110	132	86	0.022
	PIC	R	307	74	116	68	0.035
		L	337	113	104	71	0.016
	RIC	R	377	50	88	69	0.01
		L	277	114	103	71	0.016
	ACR	R	847	68	167	70	0.014
		L	753	109	160	86	0.018
	SCR	R	396	73	140	104	0.016
		L	616	109	113	112	0.014
	PCR	R	402	61	80	93	0.01
		L	415	108	85	111	0.012
	PTR	R	591	58	81	87	0.01
		L	510	119	75	90	0.013
	SS	R	364	50	87	67	0.01
		L	239	128	81	62	0.034
	EX	R	234	55	117	63	0.014
		L	379	117	139	83	0.022
	ST	R	26	59	109	62	0.016
		L	27	122	108	62	0.02
	SLF	R	595	58	87	106	0.02
		L	589	125	93	100	0.014
	SFOF	R	30	67	133	91	0.025
		L	44	112	135	91	0.022
	UC	L	29	126	123	53	0.02
	T	R	22	61	76	90	0.01
	**RD**						
	MCP	–	46	77	105	45	0.04
	PCT	–	120	82	95	43	0.043
	GCC	–	567	106	158	87	0.026
	BCC	–	1044	96	145	91	0.025
	SCC	–	636	110	76	96	0.014
	CT	R	72	81	107	49	0.04
	ML	R	62	86	90	30	0.043
		L	41	94	90	35	0.043
	SCP	R	11	82	95	51	0.048
	CP	R	249	74	104	61	0.019
		L	76	104	102	67	0.022
	AIC	R	334	67	144	82	0.03
		L	291	112	135	90	0.021
	PIC	R	417	67	109	75	0.019
		L	291	117	109	87	0.021
	RIC	R	412	50	88	69	0.014
		L	259	114	103	71	0.021
	ACR	R	989	63	141	92	0.029
		L	782	114	141	88	0.021
	SCR	R	568	73	120	109	0.041
		L	783	109	113	112	0.015
	PCR	R	422	61	81	94	0.016
		L	455	108	85	111	0.014
	PTR	R	593	50	87	69	0.014
		L	462	120	73	87	0.014
	SS	R	388	50	88	67	0.014
		L	252	132	96	58	0.027
	EC	R	622	54	109	65	0.016
		L	448	119	135	62	0.042
	ST	R	25	57	104	65	0.019
		L	12	122	102	66	0.049
	SLF	R	551	55	82	102	0.016
		L	353	127	95	102	0.035
	SFOF	R	40	67	132	92	0.036
		L	49	112	135	91	0.021
	UC	L	26	125	123	53	0.03
	T	R	21	59	74	87	0.016
CD/CU + < HC	**RD**						
	GCC	–	81	103	146	95	0.045
	BCC	–	25	105	138	101	0.049

The table contains significant clusters of voxels (at least 10 significant voxels) within gyral or subcortical white-matter tracts on the Tract-Based Spatial Statistics-derived fractional anisotropy (FA), axial diffusivity (AD), radial diffusivity (RD) and mean diffusivity (MD) skeletons between groups (CD/CU+, CD/CU- and healthy controls). Results depict white matter label, hemisphere, cluster size, peak x,y,z coordinates, and *p*-values. Key: BCC = body corpus callosum; SCC = splenium corpus callosum; CP = cerebral peduncle; PIC = posterior internal capsule; RIC = retrolenticular internal capsule; SCR = superior corona radiata; PTR = posterior thalamic radiation; SS = sagittal stratum; EC = external capsule; SLF = superior longitudinal fasciculus; PCR = posterior corona radiata; ST = stria terminalis; SFOF = superior fronto-occipital fasciculus; UC = uncinate fasciculus; ACR = anterior corona radiata; GCC = genus corpus callosum;CD = Conduct Disorder; CD/CU+ = Conduct Disorder with high levels of callous-unemotional traits; CD/CU- = Conduct Disorder with low levels of callous-unemotional traits; HC = Healthy Controls

**Fig. 4:**
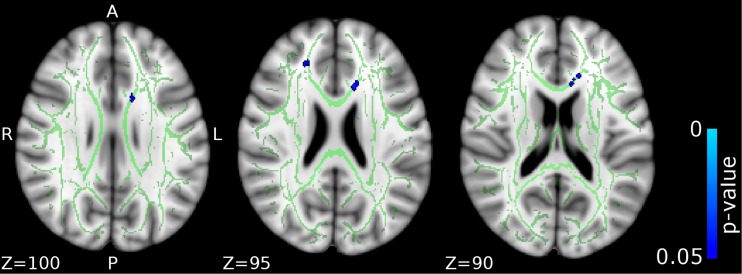
**Differences in radial diffusivity (RD) between the CD/CU+ and healthy control groups.** The figure displays regions that showed reduced radial diffusivity in adolescents with Conduct Disorder and high levels of callous-unemotional traits (CD/CU+) relative to healthy controls. The axial images are thresholded at p < 0.05, threshold-free cluster enhancement (TFCE) correction, i.e., correction for all voxels in the white-matter skeleton. Significance level of the findings is indicated by the blue color scheme (with the most significant findings shown in light blue). All findings are superimposed on the mean FA background image and the outline of the TBSS-generated white-matter tract skeleton, shown in green

The CD/CU+ group showed decreased RD (lower diffusivity) compared to HC in the genu and body of the corpus callosum and left anterior corona radiata (see Fig. 4 and Table 2).

## Analysis of Callous-Unemotional Traits as a Continuous Measure

**Fig. 5:**
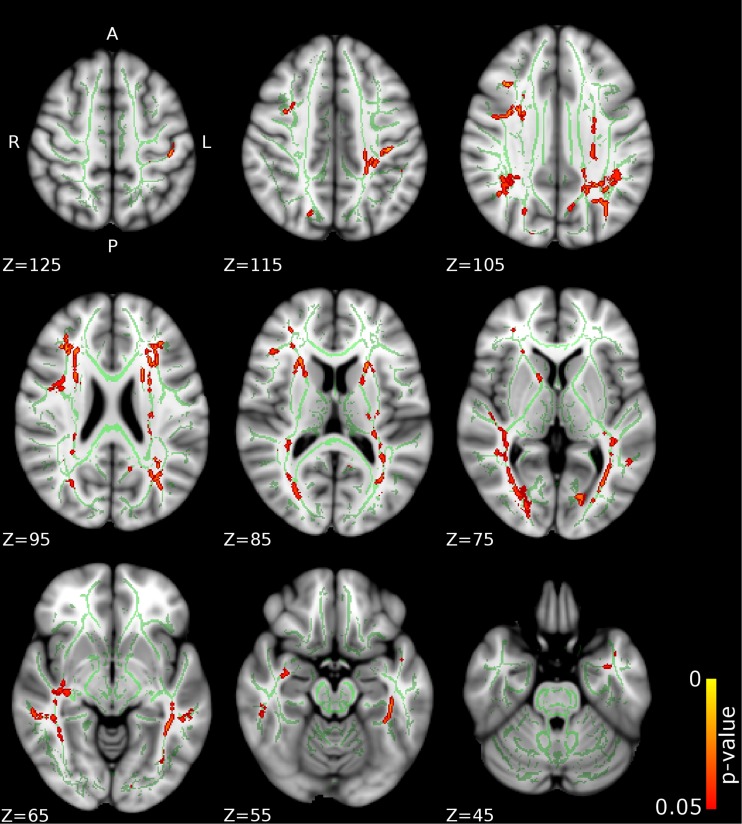
**Positive associations between callous-unemotional traits and fractional anisotropy (FA) values.** The figure depicts white matter tracts where a positive association between callous-unemotional traits and FA was observed. The axial images are thresholded at p < 0.05, threshold-free cluster enhancement (TFCE) correction, i.e., correction for all voxels in the white-matter skeleton. Significance level of the findings is indicated by the red-yellow color scheme (with the most significant findings shown in yellow). All findings are superimposed on the mean FA background image and the outline of the TBSS-generated white-matter tract skeleton, shown in green

**Fig. 6:**
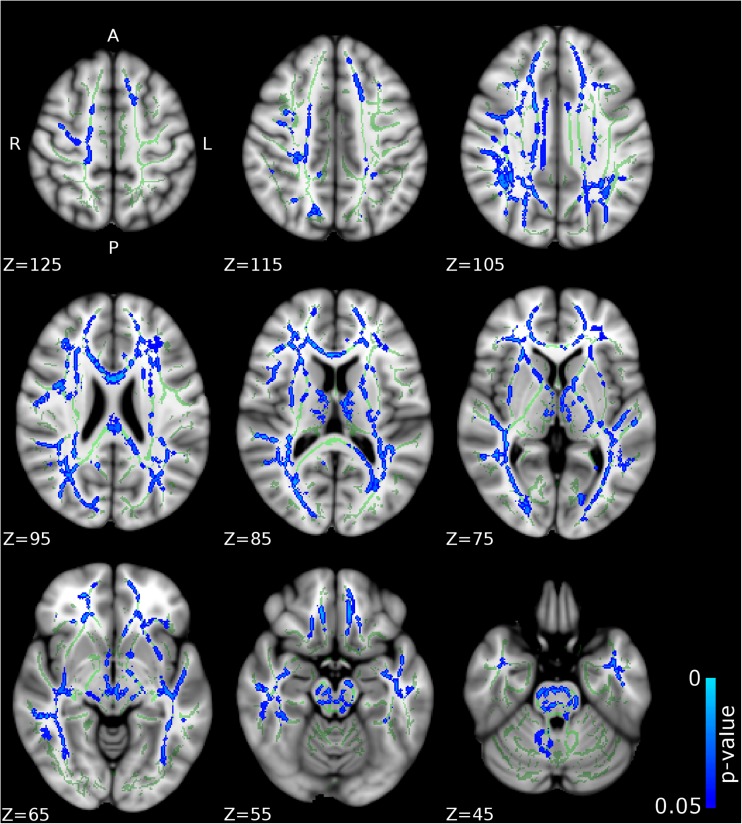
**Negative associations between callous-unemotional traits and mean diffusivity (MD) values.** The figure depicts white matter tracts where a negative association between callous-unemotional traits and MD was observed. The axial images are thresholded at p < 0.05, threshold-free cluster enhancement (TFCE) correction, i.e., correction for all voxels in the white-matter skeleton. Significance level of the findings is indicated by the blue color scheme (with the most significant findings shown in light blue). All findings are superimposed on the mean FA background image and the outline of the TBSS-generated white-matter tract skeleton, shown in green

**Fig. 7:**
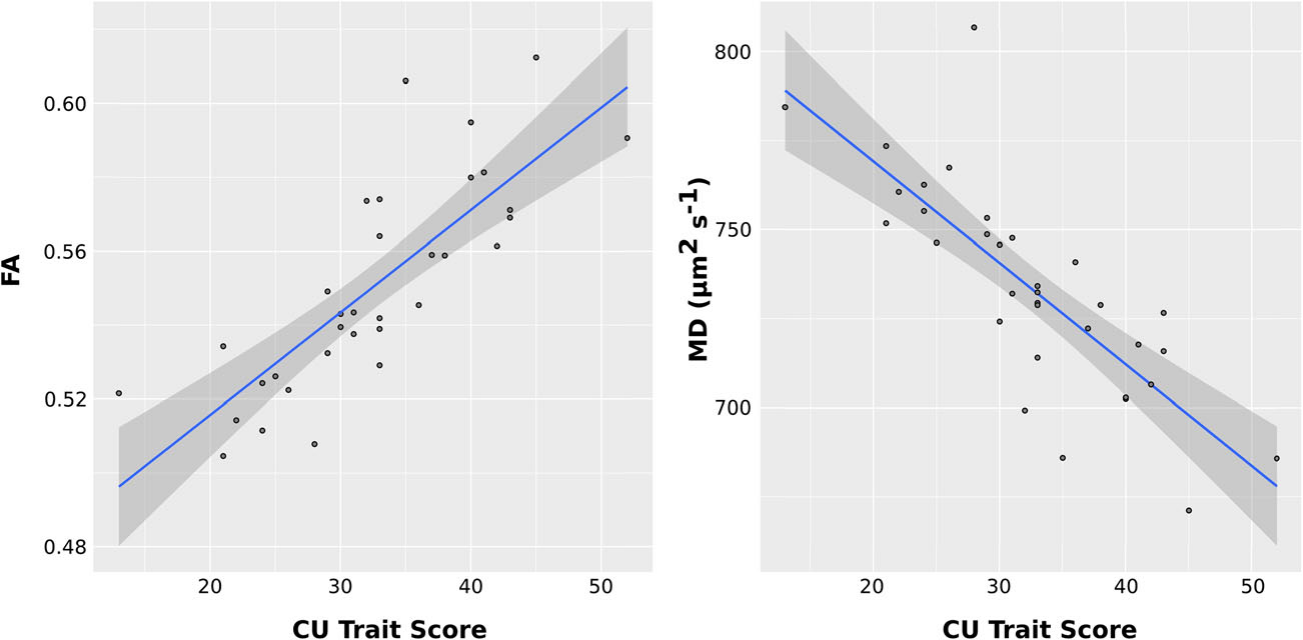
**Scatter plots of the positive associations between callous-unemotional traits and fractional anisotropy (FA) and negative associations between callous-unemotional traits and mean diffusivity (MD) values.** The scatter plots depict average FA or MD over all significant voxels reported by TBSS. The effects of age, IQ, ADHD symptoms, pubertal stage and substance abuse were removed by fitting a linear model (with no interactions) and the residuals of the model were plotted against callous-unemotional traits

Associations between callous-unemotional (CU) traits and skeletonized FA, AD, RD and MD were investigated using voxel wise GLM correcting for age, IQ, ADHD symptoms, and PDS and PESQ scores. Callous-unemotional traits showed a significant positive association with FA in the following tracts: bilateral internal capsule, bilateral external capsule, bilateral corona radiata, bilateral thalamic radiation, bilateral inferior longitudinal fasciculus (ILF) and inferior fronto-occipital fasciculus (IFOF) and bilateral superior longitudinal fasciculus (SLF) (see Figs. 5 and 7 and Table 3). Callous-unemotional traits showed a very widespread significant negative association with MD (see Figs. 6 and 7 and Table 3). Importantly, tracts where a negative association between CU traits and MD was found overlapped with white-matter tracts (listed above) where a positive association between CU traits and FA was observed.

**Table 3:** Tracts showing significant associations between callous–unemotional traits and FA, AD, RD and MD in simple regression models

	Callous-unemotional							
Simple regression	Direction	Associated tracts	Hemisphere	Cluster size	Peak x,y,z			TFCE corrected *p*-value
**FA**	Positive							
		AIC	R	164	67	143	82	0.025
			L	168	113	132	90	0.02
		PIC	L	35	114	105	86	0.042
		RIC	R	141	55	88	80	0.04
			L	187	117	93	86	0.037
		ACR	R	203	66	146	84	0.025
			L	205	113	141	87	0.021
		SCR	R	184	62	128	102	0.02
			L	265	115	102	105	0.037
		PCR	R	50	62	92	92	0.043
			L	301	115	101	105	0.037
		PTR	R	445	60	55	76	0.039
			L	479	118	58	73	0.028
		SS	R	159	54	73	68	0.042
			L	198	131	97	58	0.026
		EC	R	58	54	113	64	0.048
			L	62	118	105	89	0.041
		ST	R	38	58	107	63	0.042
		SLF	R	134	57	132	99	0.029
			L	25	120	99	107	0.05
		SFOF	L	46	112	133	91	0.02
**AD**	–	–	–	–	–			–
**RD**	–	–	–	–	–			–
**MD**	Negative	MCP	–	667	68	68	39	0.047
		PCT	–	144	89	105	48	0.026
		GCC	–	750	77	153	87	0.015
		BCC	–	1090	93	137	93	0.015
		SCC	–	454	105	86	96	0.03
		CST	R	157	86	105	38	0.022
			L	176	94	106	39	0.02
		ML	R	32	86	88	33	0.04
			L	78	97	87	37	0.033
		ICP	R	53	78	77	46	0.044
			L	17	101	83	36	0.033
		SCP	R	36	81	77	41	0.045
			L	44	96	87	45	0.04
		CP	R	179	83	107	51	0.026
			L	193	94	104	51	0.027
		AIC	R	254	67	144	81	0.018
			L	312	101	133	72	0.029
		PIC	R	75	70	122	81	0.045
			L	457	113	103	73	0.025
		RIC	R	222	55	88	80	0.007
			L	394	117	93	86	0.023
		ACR	R	792	73	153	93	0.016
			L	637	112	143	85	0.03
		SCR	R	426	73	140	103	0.017
			L	322	114	131	91	0.04
		PCR	R	388	62	92	92	0.014
			L	329	116	96	97	0.023
		PTR	R	721	55	87	81	0.007
			L	686	127	75	77	0.017
		SS	R	319	50	87	67	0.014
			L	278	128	81	62	0.025
		EC	R	317	56	122	74	0.026
			L	396	118	138	78	0.039
		CG	R	192	81	121	107	0.042
			L	100	97	153	93	0.033
		ST	R	35	57	110	61	0.028
			L	115	118	95	70	0.029
		SLF	R	551	47	82	78	0.018
			L	160	127	84	86	0.024
		SFOF	R	25	67	123	92	0.046
			L	38	111	138	94	0.038
		T	R	17	58	75	86	0.014

The reported tracts contained at least 10 significant voxels. AIC = anterior internal capsule; PIC = posterior internal capsule; RIC = retrolenticular internal capsule; ACR = anterior corona radiata; SCR = superior corona radiata; PCR = posterior corona radiata; PTR = posterior thalamic radiation; SS = sagittal stratum; EC = external capsule; ST = stria terminalis; SLF = superior longitudinal fasciculus; SFOF = superior fronto-occipital fasciculus; MCP = middle cerebellar peduncle; PCT = pontine crossing tract; GCC = genus corpus callosum; BCC = body corpus callosum; SCC = splenium corpus callosum; CST = corticospinal tract; ML = medial lemniscus; ICP = inferior cerebellar peduncle; SCP = superior cerebellar peduncle; CP = cerebral peduncle; CG = cingulate gyrus; T = tapetum

## Discussion

The key finding of the present study was that white-matter microstructural abnormalities in adolescents with CD were specifically associated with elevated callous-unemotional traits. In fact, our analysis comparing the CD group as a whole (i.e., with the CD/CU+ and CD/CU- groups combined) versus healthy controls did not yield any significant group differences. However, when the CD participants were split according to their level of CU traits, a different picture emerged. Compared to CD/CU- individuals, participants with CD/CU+ showed increased FA and reduced RD and MD (lower diffusivity) in commissural, association tracts and projection/thalamic pathways. The CD/CU+ group also showed reduced RD and MD in the left uncinate fasciculus and bilateral fornix compared to the CD/CU- group. Furthermore, the CD/CU+ group showed decreased RD compared to the HC group in the genu and body of the corpus callosum and left anterior corona radiata.

Therefore increases in FA and reductions in RD/MD in key white-matter tracts including (but not limited to) those connecting prefrontal and limbic regions (uncinate fasciculus) appeared to be specifically linked to CD with elevated callous-unemotional traits (CD/CU+). This was further confirmed by our correlational analysis whereby CU traits were treated as a continuous variable and associations with FA, AD, RD, and MD were investigated. We found a positive relationship between CU traits and FA and a negative relationship between CU traits and MD in several association tracts and projection/thalamic pathways (partially replicating our group-based findings).

Our results are in keeping with three out of four previous studies that have investigated CU traits in adolescents with CD. In line with Pape et al. (2015) and Zhang et al. (2014b), the present study found increased FA and reduced RD (lower diffusivity) in the corpus callosum of CD/CU+ compared to CD/CU- individuals. In keeping with Pape et al. (2015) and Zhang et al. (2014a), increased FA and reduced RD in the right ILF, right IFOF, left SLF, various other tracts belonging to the cortico-spinal tract (left cerebral peduncle, bilateral internal capsule), and projection/thalamic pathways (left superior and posterior corona radiate, bilateral thalamic radiation and left external capsule) was found in CD/CU+ relative to CD/CU- individuals. Furthermore, in accordance with Pape et al. (2015), Zhang et al. (2014a) and Sarkar et al. (2013) we also found reduced RD and MD (lower diffusivity) in the left UF of CD/CU+ relative to CD/CU- individuals.

However, our findings diverge from those reported by Breeden et al. (2015), who observed negative correlations between CU traits and FA values in the uncinate fasciculus and stria terminalis. It is worthy of note that Breeden et al. (2015) used a region of interest (ROI) based approach and therefore, it is not known what the direction of the correlation between CU traits and FA (and RD/AD) in other relevant tracts (other than UF and stria terminalis) might have been and therefore, whether the results of the two studies could have converged in relation to other tracts. Unlike Breeden et al. (2015), in our analysis we controlled for ADHD symptoms, pubertal stage and substance use and therefore this may explain the divergent results.

From a functional perspective, white-matter microstructure abnormalities in the corpus callosum may be related to an imbalance in interhemispheric communication between the frontal (including prefrontal, premotor, supplementary motor, and motor cortices) and parietal cortices. This could potentially play an important role in abilities such as: emotion regulation, motor coordination, motor planning, and executive functions. Imbalances in interhemispheric communication have also been related to other aspects of antisocial behavior, such as impulsivity and aggression (Lindner et al. 2016; Schutter and Harmon-Jones 2013).

We also found reduced RD and MD in CD/CU+ individuals in the left uncinate fasciculus, (connecting the orbitofrontal cortex and anterior temporal lobe, including the amygdala (Catani et al. 2013). This may contribute to impaired top-down regulation of amygdala activity by the orbitofrontal cortex in individuals with CD/CU+. This dysregulation may result in deficits in emotion regulation leading to anger outbursts or empathic impairments (e.g., responding appropriately to the emotions of others). Functional MRI studies have provided evidence for reduced amygdala responses to fearful facial expressions in children with CD/CU+ compared to controls (Jones et al. 2009; Marsh et al. 2008) - an impairment related to reduced amygdala and ventromedial prefrontal cortex activation in response to distress cues (Blair 2013). Our findings of abnormal structural connectivity in the left UF in CD/CU+ individuals could explain why this group shows impairments in learning from punishment or learning to avoid actions that have negative consequences for themselves or others (Blair 2013; Budhani and Blair 2005).

We also found increased FA and reduced RD and MD in several other association tracts including the right IFOF, left SLF and right ILF. The right IFOF is a long-range association fiber tract that connects the frontal and occipital lobes (Catani 2006). Damage to the posterior/occipital portion of the IFOF has been associated with visual neglect as a result of impaired modulation by the frontal cortex (Urbanski et al. 2008), as well as impaired facial emotion recognition (Philippi et al. 2009), face processing (Sundram et al. 2012) and empathic abilities (Parkinson and Wheatley 2014). Increased FA in the left SLF has been associated with better spatial working memory performance in typical children and adolescents (Vestergaard et al. 2011). Sundram et al. (2012) suggested that reduced microstructural integrity of the IFOF may contribute to deficits in face processing in antisocial populations, who show disproportionate deficits in fear recognition relative to other expressions (Marsh and Blair 2008).

Finally, we found increased FA and reduced RD and MD in projection/thalamic pathways (the left cerebral peduncle, bilateral internal capsule, left superior and posterior corona radiata, bilateral thalamic radiation and left external capsule). The anterior thalamic radiation comprises fibers that run between medio-dorsal thalamic nuclei and the frontal cortex and also between anterior thalamic nuclei and the anterior cingulate cortices (Catani et al. 2013). Structural and functional abnormalities in the anterior thalamic radiation have been associated with deficits in executive functions specifically related to declarative memory, such as the use of strategies during memory retrieval (Van der Werf et al. 2003).

The microstructural properties of the anterior thalamic radiation and IFOF have also been associated with individual differences in empathic abilities (experiencing emotion in response to the perceived emotions of others). Parkinson and Wheatley (2014) found that greater levels of empathic concern were associated with greater FA in association fiber tracts linking areas involved in visual and affective processing (ILF and IFOF), and areas of the limbic system (UF and anterior thalamic radiation).

### Strengths and Limitations

The present study has several important strengths, as well as limitations, that should be noted. In terms of strengths, the present study has a fairly large sample size (37 CD participants and 35 healthy controls) compared to other DTI studies published within the literature on CD (Waller et al. 2017). Accordingly, the relatively large sample size enabled us to systematically investigate the impact of callous-unemotional traits in our CD sample (with 18 CD/CU+ and 17 CD/CU- participants), and also contrast each of these groups with a relatively large group of healthy controls (*n* = 32). We were also able to examine multiple indices of white-matter diffusivity, including AD, RD and MD, which allowed us to investigate the factors that contributed to group differences in FA. We also applied stringent correction for multiple comparisons, in line with recommendations by the developers of the TBSS approach (Smith et al. 2007), so this increases the likelihood that our findings will prove to be robust and replicable. We supplemented our categorical analysis with a correlational design to examine the relationship between CU traits and FA, AD, RD and MD values and found positive correlations between CU traits and FA and negative correlations between CU traits and MD, largely replicating the findings of our group-based analyses separating the CD sample in CD/CU+ and CD/CU- groups. Due to the detailed characterization of our sample, we were able to control for various confounding variables such as comorbid ADHD symptoms, age, pubertal stage, IQ and substance use.

As a general limitation that applies to the current DTI research at large we recognise that FA differences between groups could be a result of several factors (myelination, axon density, axon diameter, membrane permeability, or even axonal orientation and fiber crossing) and that simple DTI tensor calculations as employed in our study may not conclusively link the regional abnormalities to any specific mechanism (Jones et al. 2013). Even though AD and RD and MD provide more specific information about FA, the exact physiological correlates of these measures are still under scientific scrutiny (Jones et al. 2013).

Potential limitations of the study were that participants were allocated to CD/CU+ and CD/CU- groups based on a median split of the ICU scores, rather than on the basis of independently established cut-offs. This is a common approach in the literature, which reflects the fact that no reliable cut-off scores currently exist on measures of callous-unemotional traits such as the ICU. However, our grouping approach appeared to be sensitive because it revealed important white-matter microstructure differences between the CD/CU+ and CD/CU- groups, as well as significant differences between the CD/CU+ and HC groups. We also used research diagnoses of Conduct Disorder; therefore, it would be interesting to replicate the present research design using clinically-assessed participants. Nevertheless, the sample was well-characterized and diagnostic information was available from the participants themselves and the majority of their parents or carers. Another potential limitation concerns the fact that our CD-CU+ sample might have had a higher use of medication compare to CD-CU- and controls and this could have contributed to our results. Unfortunately we were not able to measure medication use in our sample and therefore could not control for this variable in our analysis.

A further limitation of the study is that CU traits were assessed using the self-report version of the ICU questionnaire. However, we note that over 130 studies have used self-report measures of CU or psychopathic traits such as the ICU (Frick et al. 2014) and there is still no consensus whether parents, teachers or the young people themselves are better informants about the presence or absence of CU traits and the guidance in the DSM-5 is that information from all three sources can be used in clinical settings (American Psychiatric Association 2013).

### Conclusion

In summary, the present study shows that white-matter microstructural alterations in key white-matter tracts (body and splenium of the corpus callosum, right ILF, right IFOF, left SLF, left cerebral peduncle, bilateral internal capsule, left superior and posterior corona radiate, bilateral thalamic radiation and left external capsule, left uncinate fasciculus and fornix) previously implicated in socioemotional development were observed specifically in adolescents with CD and elevated callous-unemotional traits, rather than in those with CD and lower levels of callous-unemotional traits. These findings provide support for subtyping CD according to the presence or absence of callous-unemotional traits, and add to the evidence base suggesting that these two variants of CD may have different etiologies and neurodevelopmental origins. Advancing our understanding of the neurobiological basis of CD and its subtypes is important as CD is frequently a precursor to adult antisocial personality disorder (Loeber et al. 2002). A better understanding of the etiology of CD might enable the development of more specific treatments and interventions which could prevent the transition from CD to antisocial personality disorder and psychopathy.
